# Habitat Quality Assessment Within Expanded Ranges of Dengue Vectors Using a Composite Index Scale

**DOI:** 10.1002/ece3.72387

**Published:** 2026-01-24

**Authors:** Muhammad Naeem, Lei Zhu, Nawaz Haider Bashir, Maryam Riasat, Wenbo Li, Huanhuan Chen

**Affiliations:** ^1^ College of Biological Resource and Food Engineering Qujing Normal University Qujing China; ^2^ Faculty of Engineering and Applied Sciences, Department of Zoology Riphah International University, Faisalabad Campus Faisalabad Pakistan

**Keywords:** *Aedes aegypti*, *Aedes albopictus*, *composite index scale*, habitat quality, vulnerability

## Abstract

As climate change drives shifts in species distributions, understanding the habitat quality within expanded ranges (defined as newly suitable areas (km^2^) under future climate scenarios compared to the past baseline, identified using a ≥ 0.5 suitability threshold) remains a critical gap in accurately assessing disease vector risk. Although many studies focus on the geographic range expansion of *Aedes* species under future climate scenarios, it remains unclear whether these expanded ranges possess the habitat quality necessary for stable mosquito populations. Using MaxEnt modeling, we projected habitat suitability across historical and future climate scenarios (SSP1‐4) and applied a Composite Index Scale (CIS) to quantify habitat quality within Asia. Results indicate that, in the past, habitat vulnerability for *Aedes aegypti* and *A. albopictus* was 1,698,972 km^2^ and 1,328,577 km^2^, respectively. Future projections suggest that vulnerable suitable habitat areas for *A. aegypti* will increase to between 1,991,596 km^2^ and 2,220,554 km^2^ by the end of the 21st century. In contrast, the expected rise in suitable areas for *A. albopictus* will be between 1,589,240 km^2^ and 1,734,846 km^2^. We propose the CIS, which incorporates normalized bioclimatic suitability, habitat suitability changes, niche breadth, niche position, and range shifts, as a refined measure to better quantify habitat quality. Findings reveal that 12 of the 16 scenarios showed improved habitat quality for *A. aegypti* , highlighting an elevated risk of dengue transmission. For *A. albopictus* , 10 of the 16 scenarios demonstrated high vulnerability due to favorable habitat quality as predicted by the CIS. A significant correlation was found between CIS values and the number of vulnerable pixels with values ≥ 0.5, highlighting the effectiveness of the CIS as a robust indicator for habitat quality assessment and associated risk.

## Graphical abstract

Using MaxEnt and a novel Composite Index Scale (CIS), we quantified habitat quality for *Aedes aegypti* and *A. albopictus* across historical and future (SSP1–4) climate scenarios in Asia. We found that vulnerable suitable habitat areas for *A. aegypti* and *A. albopictus* will expand by up to ~2.22 million km^2^ and ~1.73 million km^2^, respectively, with 12 of 16 and 10 of 16 scenarios showing high vulnerability. CIS values were strongly correlated with the number of pixels ≥ 0.5, demonstrating its effectiveness as a robust predictor of dengue vector risk.

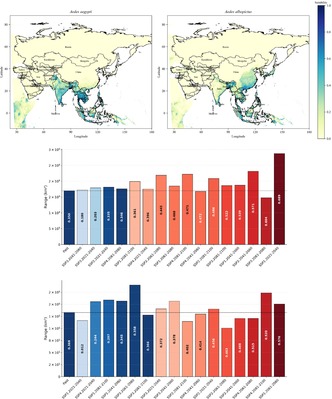

## 1 Introduction

Global climate change is affecting species distributions worldwide, leading to shifts, expansions, or contractions in species ranges (Evans and Jacquemyn 2022; Harrison et al. 2024; Wiens and Zelinka 2024). These range shifts and expansions (defined as increases in suitable habitat relative to the past baseline (areas predicted as suitable with a logistic probability ≥ 0.5)) pose increased risks to communities, especially when they involve disease vectors such as those for dengue. Dengue is the most common arboviral disease globally, with cases rising over the past 50 years alongside the geographic expansion of its primary vectors, *Aedes aegypti* and *Aedes albopictus* (Aliaga‐Samanez et al. 2024; Colón‐González et al. 2018; Li et al. 2023; Morin et al. 2013; Murray et al. 2013; Wu et al. 2018). This endemic disease is widespread across Asia and has also reached the Middle East (Abbasi 2025a; Abbasi et al. 2025; Altassan et al. 2019; Lee and Farlow 2019). However, the quality of habitat within these expanded ranges remains uncertain, as not all expanded areas are likely to support stable vector populations capable of sustaining dengue transmission.

Temperature and precipitation are key factors in determining habitat quality for the establishment of mosquito vectors that transmit diseases. *Aedes* species, for instance, are particularly sensitive to climatic conditions, and even a slight increase in temperature can elevate disease transmission rates (Andriamifidy et al. 2019; Portilla Cabrera and Selvaraj 2020). Consequently, the habitat quality of these vectors is closely linked to bioclimatic conditions. To assess the quality of expanded ranges effectively, it is important to evaluate bioclimatic suitability alongside ecological niche parameters, such as niche breadth and niche position, for dengue mosquito vectors. These niche characteristics serve as indicators of species sensitivity to climate change and help predict climate‐driven habitat shifts (Evans and Jacquemyn 2022; Thuiller et al. 2005). Additionally, factors like range shifts and habitat change are also crucial in detecting habitat quality within expanded ranges.

Numerous studies have examined dengue mosquito vectors, with a primary focus on Asia, as the region accounts for 70% of global dengue cases (Rocklöv et al. 2016; World Health Organization 2023). However, these studies have largely overlooked habitat quality within the expanded ranges of these species. For instance, a recent study assessed the future risk of dengue mosquito vectors under various scenarios without considering the habitat quality (Jing et al. 2024). Although MaxEnt species distribution modeling was used to predict the geographic range of these vectors in specific regions and scenarios (Jácome et al. 2019), it does not account for whether these projected future ranges will provide suitable habitat conditions to support stable mosquito populations capable of disease transmission.

Knowledge about habitat quality within the expanded ranges of species predicted by habitat suitability modeling remains limited. Although a previous study assessed habitat quality for *A. aegypti* on the basis of environmental suitability, this analysis was restricted to urban and local areas and did not address the quality of habitats in expanded future ranges (Misslin and Daudé 2017). Therefore, the aim of this study is to determine whether habitat quality assessments within expanded ranges can more accurately predict dengue risk under various future climate scenarios. To achieve this aim, we address the following research questions: Does habitat quality within the expanded ranges of *A. aegypti* and *A. albopictus* reliably predict dengue risk under future climate scenarios? Can the Composite Index Scale (CIS) identify high‐risk areas within expanded ranges that might benefit from targeted public health interventions?

## 2 Data and Methods

### 2.1 Data Collection and Spatial Rarefaction

**FIGURE 1:**
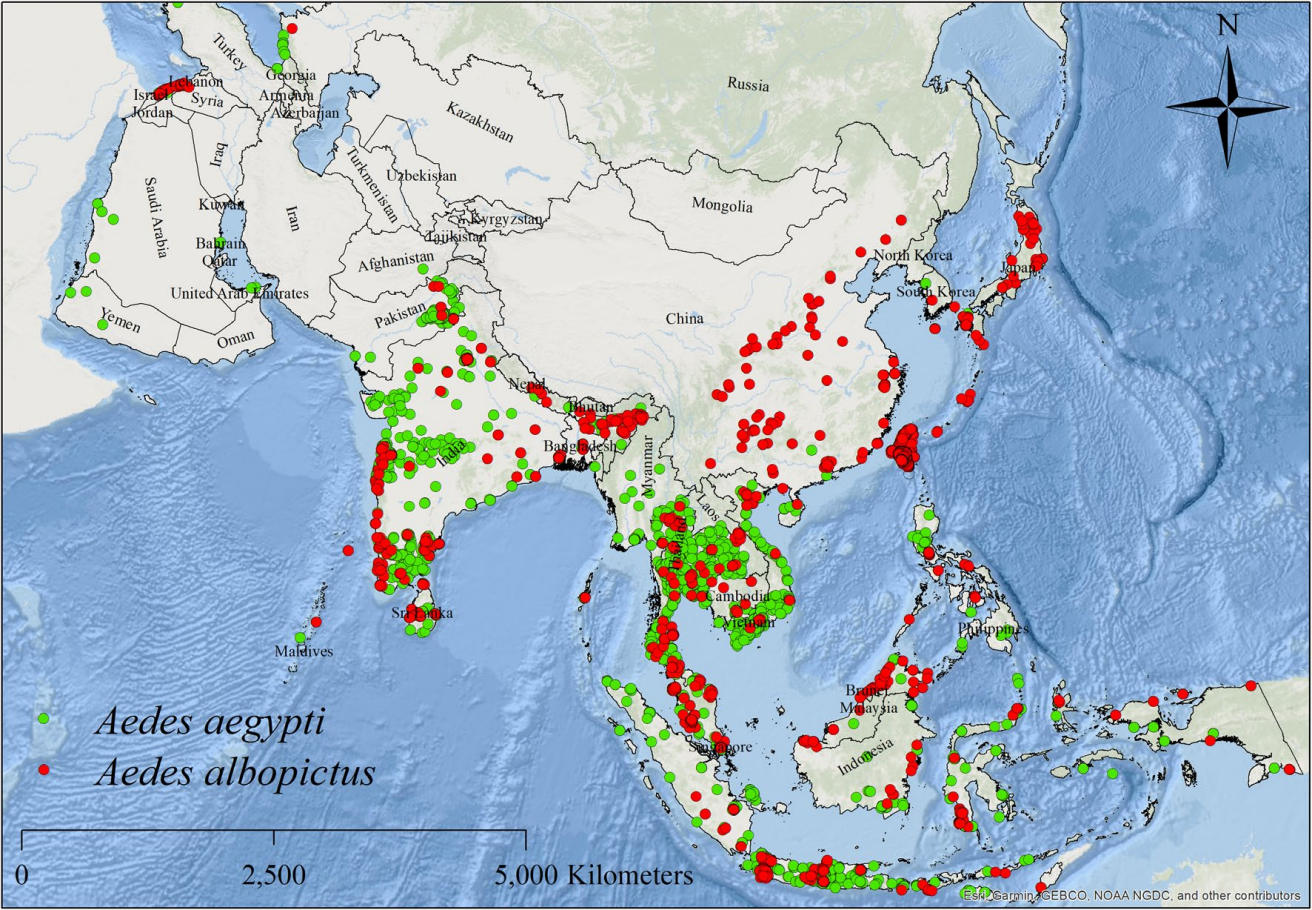
Spatial distribution of collection sites for *Aedes aegypti* and *Aedes albopictus* within Asian countries.

A total of 29,300 collection records for *A. aegypti* and *A. albopictus* were obtained from the Global Biodiversity Information Facility (GBIF, http://www.gbif.org) in October 2024. Of these, 13,065 records pertained to *A. aegypti* , whereas 16,235 records were for *A. albopictus* . Additionally, some collection data were gathered from field surveys conducted in Pakistan between 2022 and 2024 (Table S1). To ensure spatial independence and minimize biases from clustered data, the collection records were spatially rarefied using ArcToolbox in ArcMap v. 10.0, retaining only those records separated by a distance of greater than 10 km (Figure 1).

The 10 km threshold was chosen because it approximates the spatial resolution of the environmental predictor variables (5 arc‐minutes ≈ ~9.2 km at the equator), thereby aligning occurrence density with model scale and reducing spatial autocorrelation. This distance has also been widely used in previous SDM studies of mosquitoes and other insect taxa. After thinning, the dataset was reduced from 29,300 raw records (13,065 *A. aegypti* ; 16,235 *A. albopictus* ) to 721 spatially independent records (536 *A. aegypti* ; 185 *A. albopictus* ).

### 2.2 Species Distribution Modeling

To assess the potential impact of future climate change on dengue vector distributions, we employed a maximum entropy species distribution modeling approach using MaxEnt v. 3.4.4. Bioclimatic data for different time periods were downloaded from WorldClim (www.worldclim.org), covering the historical period from 1970 to 2000 (past) and projections for 2021 to 2100 (future). The future projections included four socio‐economic pathways (SSPs: SSP1, SSP2, SSP3, and SSP4), each subdivided into four time periods: 2021–2040, 2041–2060, 2061–2080, and 2081–2100. Thus, a total of 17 scenarios were generated for each species, consisting of one past and 16 future scenarios derived from global climate models (GCMs) of ACCESS‐CM2. Future climate projections were based on the ACCESS‐CM2 model, the official Australian contribution to CMIP6 (O'Kane et al. 2008). We selected ACCESS‐CM2 because it has been shown to reproduce precipitation and temperature patterns in China with high accuracy (Wang et al. 2021). In addition, downscaled climate datasets derived from ACCESS‐CM2 are publicly available and facilitate its application in regional‐scale ecological modeling. For the modeling, six non‐correlated variables (*r* < 0.9) were selected from the 19 bioclimatic variables. These included BIO2 (mean diurnal range), BIO6 (minimum temperature of coldest month), BIO11 (mean temperature of coldest quarter), BIO13 (precipitation of wettest month), BIO15 (precipitation seasonality), and BIO16 (precipitation of wettest quarter). Pearson correlation coefficients among the 19 variables were calculated in ArcGIS v10.0, and only those with *r* < 0.9 were retained.

### 2.3 Vulnerability Assessment On the Basis of Habitat Distribution

The MaxEnt modeling utilized 75% of the collection sites as training data, with the remaining 25% designated for testing. All default settings were maintained during the modeling process (Phillips et al. 2006; Wang et al. 2024). To evaluate the contribution of bioclimatic factors to the distribution of dengue mosquito vectors, we performed a jackknife test within the MaxEnt model. Model accuracy was assessed using receiver operating characteristic curves (AUC), with values exceeding 0.8 indicating excellent model performance and true skilled statistics. A total of 34 models were created: 17 for *A. aegypti* and 17 for *A. albopictus* . The outputs, generated in ASCII format from MaxEnt, were analyzed using R software v. 4.3.3, utilizing the “raster” and “classInt” packages. We calculated suitable ranges in square kilometers and converted all ASCII layers into binary maps on the basis of natural breaks threshold values. For consistency in vulnerability assessment, we defined “vulnerable pixels” as grid cells with predicted habitat suitability values ≥ 0.5 in MaxEnt, a threshold commonly used to represent areas of medium‐to‐high suitability where species establishment is most likely. The vulnerability of each species was therefore assessed in terms of the total area (km^2^) occupied by vulnerable pixels under each scenario and time period.

### 2.4 Habitat Quality Assessment Using Composite Index Scale (CIS)

Traditional species distribution modeling approaches, such as MaxEnt, primarily quantify habitat suitability by estimating the probability of species occurrence across a given geographic space. However, these models are unable to assess habitat quality, particularly within newly suitable areas identified under future scenarios. To address this limitation, we developed a Composite Index Scale (CIS) on the basis of five key variables associated with habitat suitability for dengue mosquito vectors: bioclimatic suitability, habitat suitability change, niche breadth, niche position, and range shift in future scenarios (Tables S2, S3). This framework allows us to differentiate between mere range expansion and high‐quality habitats capable of sustaining stable *Aedes* populations, thereby improving the accuracy of dengue risk prediction. Bioclimatic suitability values were extracted from MaxEnt outputs using ArcGIS software. Habitat suitability change was assessed by comparing past and future habitat suitability values, whereas range shifts were determined by analyzing suitable maps of past and future scenarios. Niche breadth and niche position were calculated using Levin's niche breadth index and the methodology outlined by Sheth (Feinsinger et al. 1981; Sheth 2014).

To optimize the weights for each factor in the CIS calculation, we initially employed a Genetic Algorithm (GA), an evolutionary optimization method that navigates complex solution spaces (Scrucca 2013). The GA optimization was evaluated using the Spearman correlation between CIS values and the habitat‐quality indicator (number of pixels ≥ 0.5). In our initial comparison, GA‐derived weights did not outperform equal weighting (*ρ* = 0.197 vs. 0.313). To ensure robustness, we compared equal weighting against GA‐derived and randomized weight sets across species and thresholds. Results were highly consistent: Spearman ρ between equal‐ and GA‐weighted CIS = 0.75 (median; 0.60–0.86), and between equal‐ and randomized‐weighted CIS = 0.86 (median; 0.83–0.90; typical 95% ranges 0.63–0.97). These findings indicate that CIS is not sensitive to the specific choice of weighting scheme; therefore, we retain equal weights for parsimony and interpretability. The CIS was calculated using equal weights by the following equation:

$$\mathrm{CIS}i=0.2\times \left(\mathrm{BS}+\mathrm{HSC}+\mathrm{NB}+\mathrm{NP}+\mathrm{RS}\right)$$

Here, CIS is composite index scale of species *i*, BS is bioclimatic suitability, HSC is habitat suitability change, NB is niche breadth, NP is niche position and RS is range shift. Before using this equation, all the variables were normalized using following equation:

$$\text{Normalized value}=\frac{X-X\min\;}{X\max-X\min}$$

where X represents the raw value of any parameter and Xmin is minimum value, and Xi is maximum value of that parameter across the entire range.

To further strengthen the robustness of the CIS, we re‐implemented a five‐fold cross‐validation procedure in MaxEnt and re‐ran the model. The dataset was randomly partitioned into five equal subsets; in each iteration, four subsets were used for training and the remaining one for testing until all subsets had been tested. Model performance was evaluated using the mean and standard deviation of the area under the curve (AUC) and true skill statistic (TSS). CIS values were then recalculated for each fold, allowing us to assess the stability of the index across independent test partitions.

## 3 Results

### 3.1 Evaluation of MaxEnt Model and Contribution of Variables

The MaxEnt modeling accuracy was high across all scenarios for both dengue mosquito vectors, *Aedes aegypti* and *Aedes albopictus* . The mean area under the curve (AUC) for the training data was 0.93 ± 0.001 for *A. aegypti* and 0.95 ± 0.001 for *A. albopictus* , with similar values for the test data. Similarly, the evaluation was also based on true skill statistic (TSS) values, which were 0.797 ± 0.032 for *A. aegypti* and 0.786 ± 0.026 for *A. albopictus* . Jackknife analysis identified key bioclimatic factors influencing species distribution. For *A. aegypti* , bio11 (mean temperature of coldest quarter) contributed the most in 7 of the 17 output models, with a contribution range of 24.5% to 42%. Bio13 (precipitation of wettest month) was the top contributor in 6 models, with contributions ranging from 22.7% to 30.5%, whereas in the remaining four models, bio6 (minimum temperature of coldest month) was the highest contributor, with a contribution range of 24.6% to 35.8%. For *A. albopictus* , bio16 (precipitation of wettest quarter) was the primary contributor in 9 models, with contributions ranging from 30.7% to 47%, and bio13 was also significant in 8 models, with contributions ranging from 29.2% to 59.5%.

### 3.2 Habitat Vulnerability and Species Distribution

**FIGURE 2:**
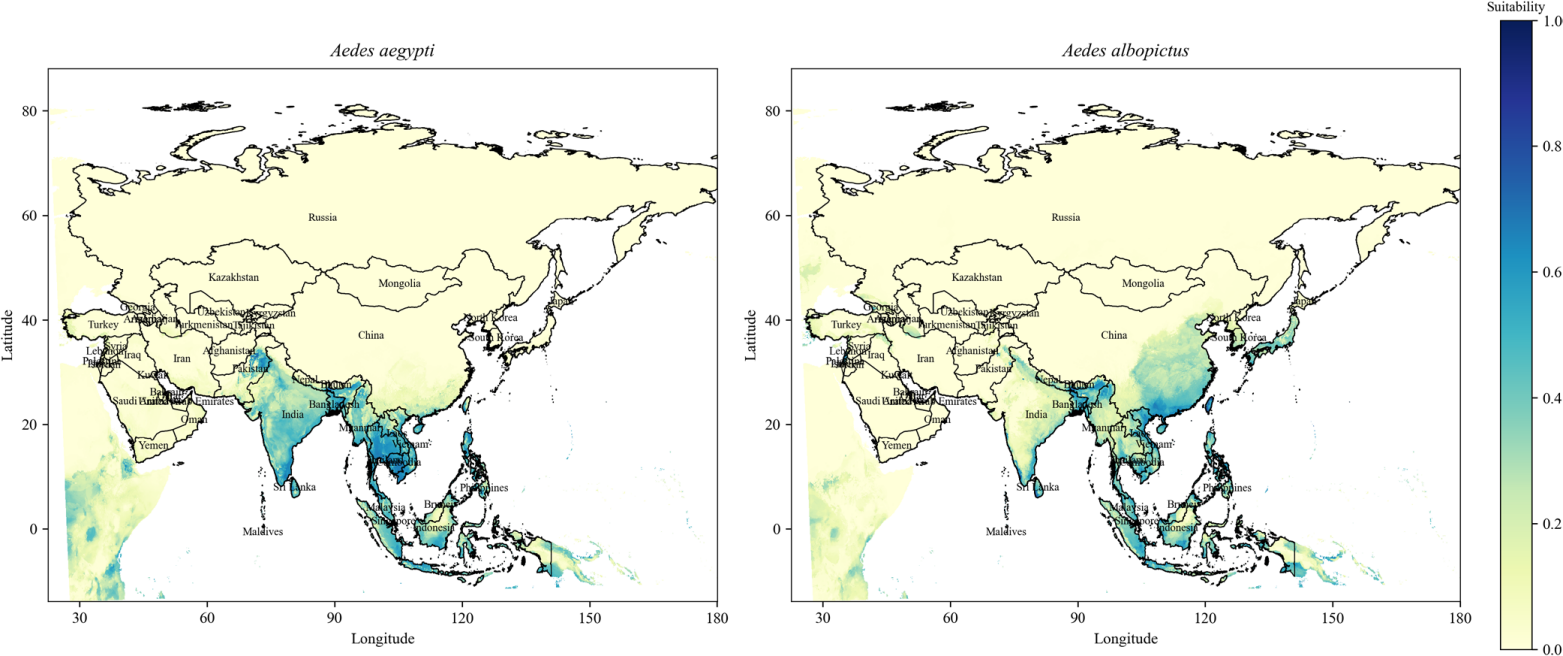
Habitat suitability areas of *Aedes aegypti* and *Aedes albopictus* in Asia under the Past scenario. Light yellow represents lower suitability, whereas dark blue represents higher suitability.

**FIGURE 3:**
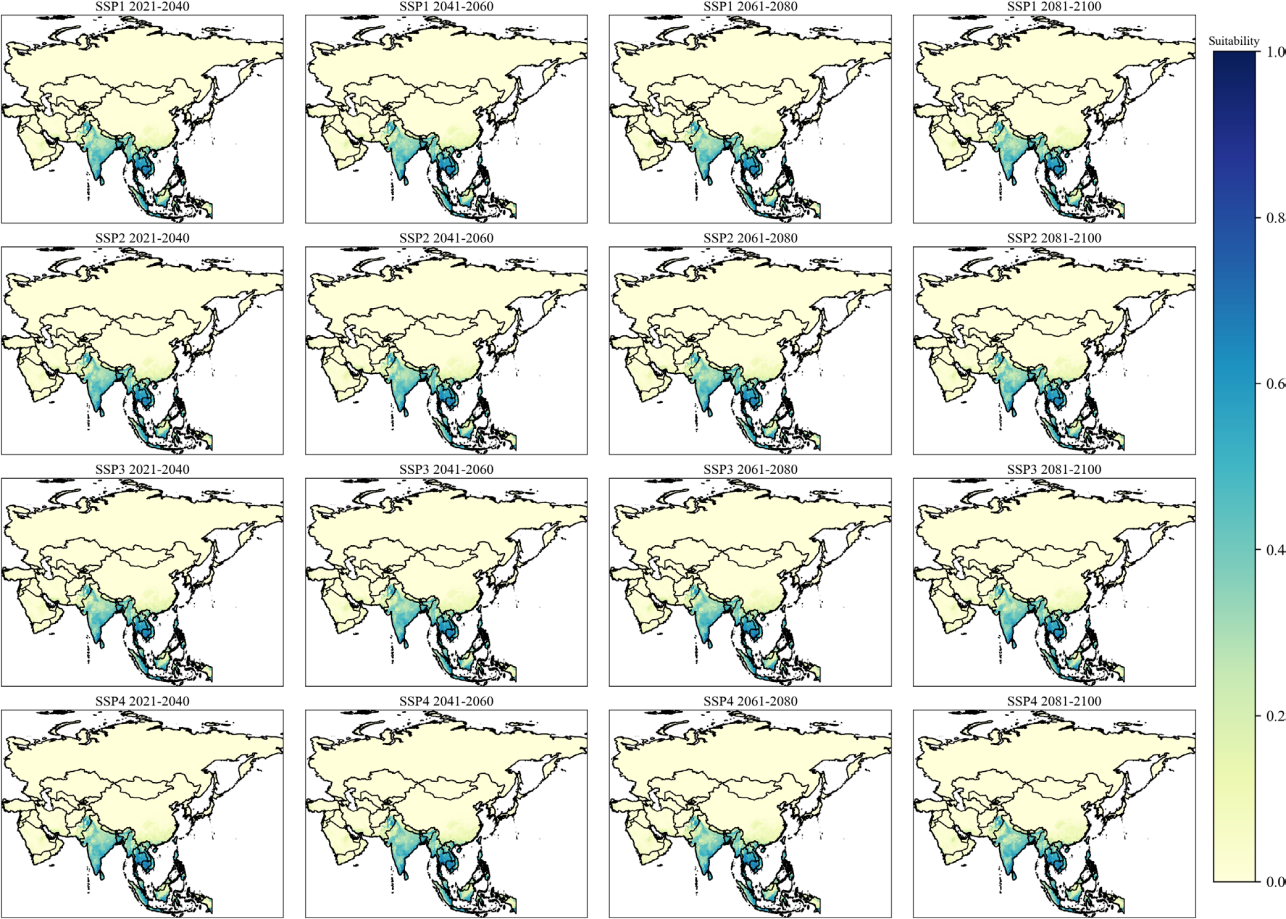
Habitat suitability of *Aedes aegypti* in Asia during future scenarios. Light yellow represents lower suitability, whereas dark blue represents higher suitability.

**FIGURE 4:**
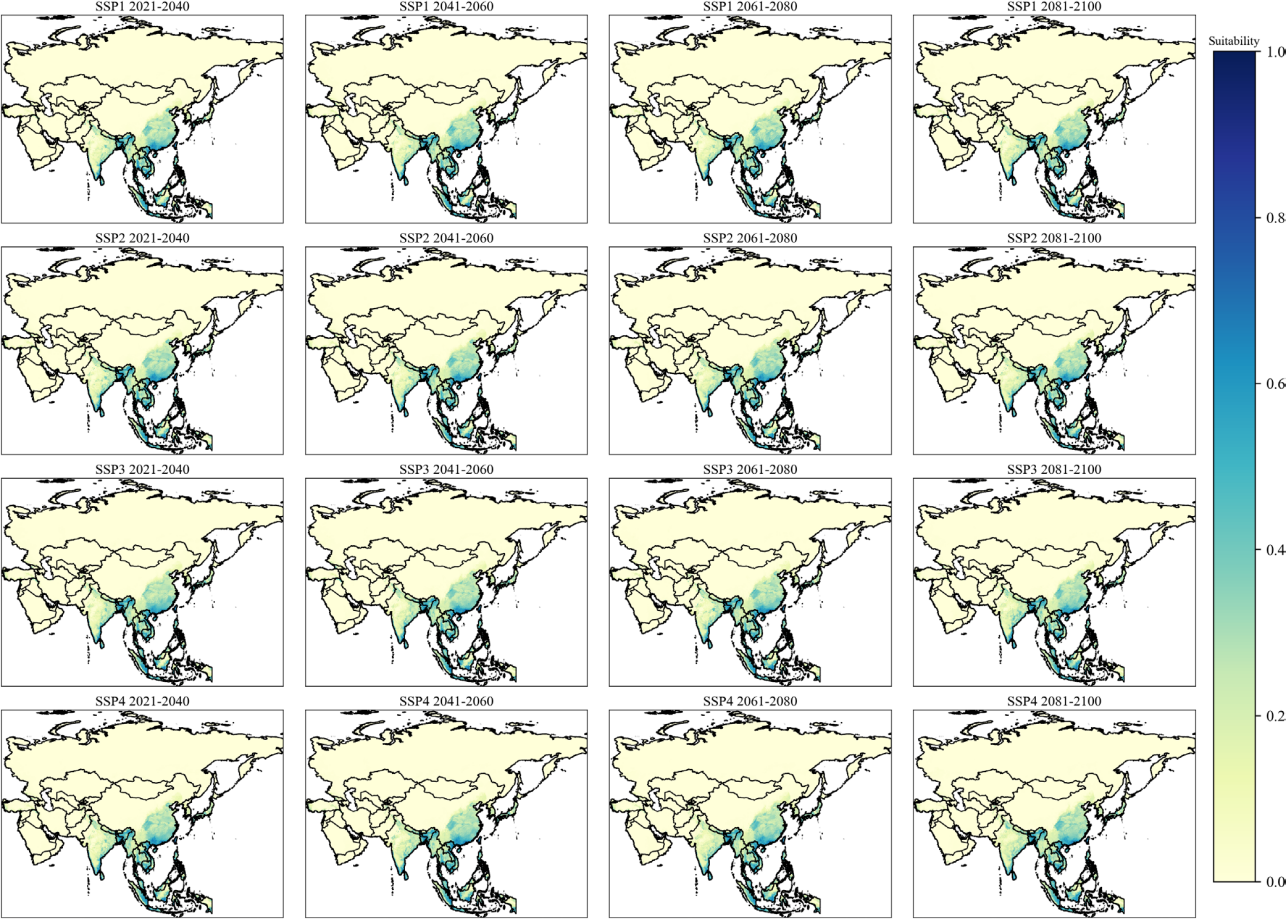
Habitat suitability of *Aedes albopictus* in Asia during future scenarios. Light yellow represents lower suitability, whereas dark blue represents higher suitability.

The MaxEnt species distribution modeling approach revealed that habitat vulnerability for the spatial distribution of *A. aegypti* and *A. albopictus* spanned 1,698,972 km^2^ and 1,328,577 km^2^, respectively, in the past (Table 1). Projections indicate that the suitable area for *A. aegypti* will increase to between 1,991,596 km^2^ (SSP1) and 2,220,554 km^2^ (SSP4) by the end of the 21st century (2081–2100) (Table 1). Similarly, suitable areas for *A. albopictus* are expected to rise from 1,328,577 km^2^ to between 1,589,240 km^2^ (SSP1) and 1,734,846 km^2^ (SSP4) during the same period (Table 1). The spatial distribution of habitat vulnerability for both species is illustrated in Figures 2, 3, and 4.

**TABLE 1:** Average suitable areas covered by *Aedes aegypti* and *A. albopictus* in whole Asia during past and future scenarios (SSP1–SSP4) in the 21st century.

Species	Time periods	Past	Area (km^2^) in different SSP scenarios			
			SSP1	SSP2	SSP3	SSP4
*A. aegypti*	Past	1,698,972				
	2021–2040		2,873,981	1,811,497	1,790,488	1,751,448
	2041–2060		1,874,144	2,315,260	1,720,328	1,677,619
	2061–2080		1,845,788	2,189,973	1,475,631	1,758,318
	2081–2100		1,991,596	2,086,009	1,860,839	2,220,554
*A. albopictus*	Past	1,328,577				
	2021–2040		1,163,401	1,400,898	1,405,090	1,555,096
	2041–2060		1,566,950	1,206,039	1,564,160	1,296,902
	2061–2080		1,504,339	1,006,331	1,899,551	1,207,510
	2081–2100		1,589,240	1,144,021	1,275,972	1,734,846

A total of 31 Asian countries were identified as vulnerable in both past and future scenarios regarding the distribution of *A. aegypti* and *A. albopictus* (Table 2). For *A. aegypti* , 16 countries showed suitable habitat ranges and the potential threat of dengue mosquito vectors in the past. This number is projected to increase to 17 countries under the SSP1 scenario and 18 countries under the SSP2, SSP3, and SSP4 scenarios by the 21st century. The most vulnerable country for *A. aegypti* in the past was Thailand, with a suitable habitat area of 401,447 km^2^, remaining the most vulnerable under the SSP2 scenario. However, in future scenarios (SSP1, SSP2, SSP3, and SSP4), India is projected to be the most vulnerable, with a suitable area of upto 442,568 km^2^ (Table 2).

**TABLE 2:** Vulnerability of area (km^2^) of different Asian countries for the spatial distribution of *A. aegypti* and *A. albopictus* during past and future scenarios.

Country	*Aedes aegypti* area (km^2^) during different SSP scenarios					*Aedes albopictus* area (km^2^) during different SSP scenarios				
	Past	SSP1	SSP2	SSP3	SSP4	Past	SSP1	SSP2	SSP3	SSP4
Bangladesh	70,106	44,661	33,348	21,666	20,595	75,341	81,827	79,906	102,226	98,500
Bhutan	1157	2045	1955	1698	1698	6314	6251	5382	6588	6557
Brunei	0	0	0	0	0	1751	1951	1726	1599	1645
Cambodia	175,011	169,368	170,324	172,622	169,323	35,481	45,714	26,708	46,142	44,658
China	21,602	32,310	29,167	24,455	27,275	279,701	265,131	231,986	264,220	261,217
Georgia	0	0	0	0	0	1869	17	0	830	0
India	299,488	442,568	432,735	346,528	389,368	197,487	264,117	202,644	265,654	229,175
Indonesia	210,748	402,723	397,040	283,035	322,697	274,109	302,366	248,444	351,685	324,535
Iran	0	151	194	65	237	86	267	258	496	323
Iraq	0	0	0	0	0	0	1943	923	2665	574
Israel	0	0	0	0	0	6347	6434	6037	5792	5385
Japan	0	0	0	0	0	18,985	19,706	15,693	23,009	18,310
Laos	57,286	67,012	62,886	50,878	48,865	18,186	30,100	18,608	24,334	26,440
Lebanon	0	0	0	0	0	2228	3908	3861	3969	4061
Malaysia	26,576	59,588	61,352	43,700	48,848	52,320	72,793	57,473	70,970	69,462
Maldives	0	0	0	0	0	9	0	0	0	0
Myanmar	90,459	114,834	109,884	77,933	95,106	50,583	27,909	23,851	35,571	29,636
Nepal	1873	2703	2601	799	412	4516	5182	4281	5666	4882
Oman	0	0	62	83	168	0	86	65	86	108
Pakistan	94,722	97,259	94,265	74,701	76,989	2842	1701	1237	2081	1830
Palestina	0	0	0	0	0	1813	2781	2454	2465	2202
Philippines	95,886	119,809	107,359	87,321	103,673	82,524	85,334	66,126	91,594	85,193
Russia	0	0	0	0	0	0	0	65	90	151
Saudi Arabia	86	0	0	0	0	0	0	0	0	0
Singapore	0	382	474	354	315	358	494	484	465	494
South Korea	0	0	0	0	0	0	80	0	74	262
Sri Lanka	22,794	39,236	43,904	34,847	42,678	19,378	24,914	16,571	21,890	25,068
Syria	0	0	0	0	0	0	1642	622	524	750
Thailand	401,447	414,044	418,563	373,159	375,348	30,397	48,933	36,498	55,906	42,697
Turkey	0	0	0	0	0	101	368	369	613	1066
Vietnam	129,729	137,686	134,573	117,978	128,389	128,075	119,303	102,331	114,103	127,815

For *A. albopictus* , 25 countries demonstrated suitable habitat ranges in the past, with vulnerability projected to increase to 28 countries under SSP1 and SSP4 scenarios, 27 countries under SSP2, and 29 countries under SSP3 by the 21st century. China was the most vulnerable country in the past, with a suitable habitat area of 279,701 km^2^, whereas Indonesia is projected to have the highest vulnerability in future scenarios, with suitable areas ranging from 248,444 km^2^ to 351,685 km^2^ (Table 2).

### 3.3 Quality Assessment of Habitat Using the Composite Index Scale (CIS)

The assessment of habitat quality using the Composite Index Scale (CIS) revealed varying quality for both species. The range of CIS values for *A. aegypti* was found to be between 0.180 and 0.689, whereas for *A. albopictus* , the range was from 0.012 to 0.576. Here, lower values indicate poor habitat quality, whereas higher values indicate better quality.

**FIGURE 5:**
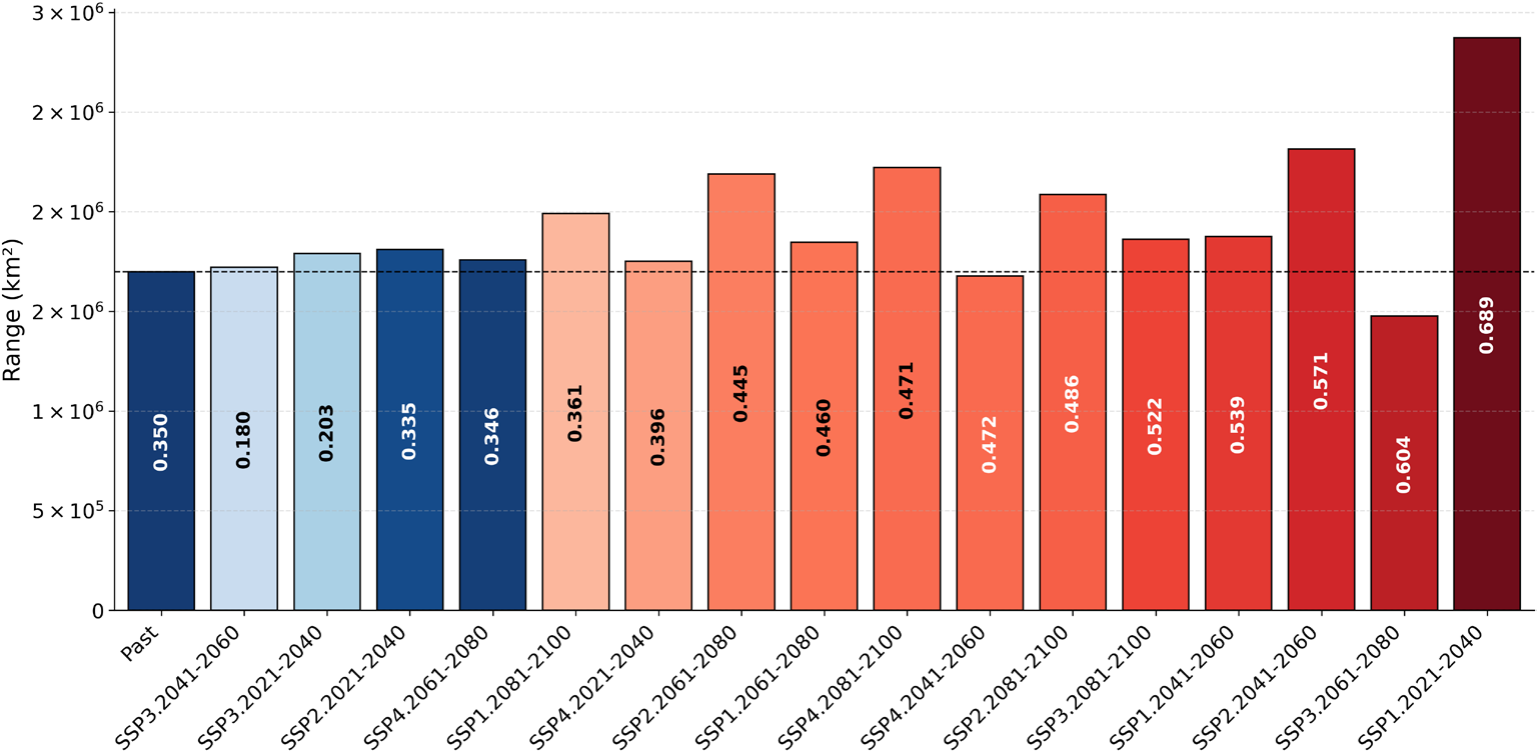
Range (km^2^) of *Aedes aegyhpti* under past and future climate scenarios. The bar heights represent the total estimated range area, whereas the numeric labels within the bars indicate the corresponding Composite Index Scale (CIS) values. Bars are ordered with the Past scenario first, followed by all future scenarios sorted by ascending CIS values. Bar colors are mapped relative to the Past CIS threshold: Blue shades denote CIS values less than or equal to the Past CIS, and red shades denote CIS values greater than the Past CIS, with darker tones indicating values farther from the threshold (higher CIS = higher risk). A thin dashed horizontal line marks the Past range to facilitate comparison with future scenarios.

Comparison of CIS values between the past and 16 future scenarios showed that 12 scenarios exhibited improved habitat quality, suggesting greater potential for dengue spread by *A. aegypti* . Specifically, this included scenarios from SSP1 across all time periods (2021–2040, 2041–2060, 2061–2080, 2081–2100), as well as SSP2 across three time periods (2041–2060, 2061–2080, 2081–2100), SSP3 across two time periods (2061–2080, 2081–2100), and SSP4 across three time periods (2021–2040, 2041–2060, 2081–2100) (Figure 5). The remaining four future scenarios displayed poor habitat quality on the basis of CIS values that were lower than the past CIS value of 0.350. These scenarios are SSP2 (2021–2040), SSP3 (2021–2040 and 2041–2060), and SSP4 (2061–2080), all of which showed CIS values ranging from 0.180 to 0.346 (Figure 5). Although MaxEnt modeling predicted an expansion of suitable ranges under these scenarios, the lower CIS values indicated that the habitat quality remained poor (Figure 5).

**FIGURE 6:**
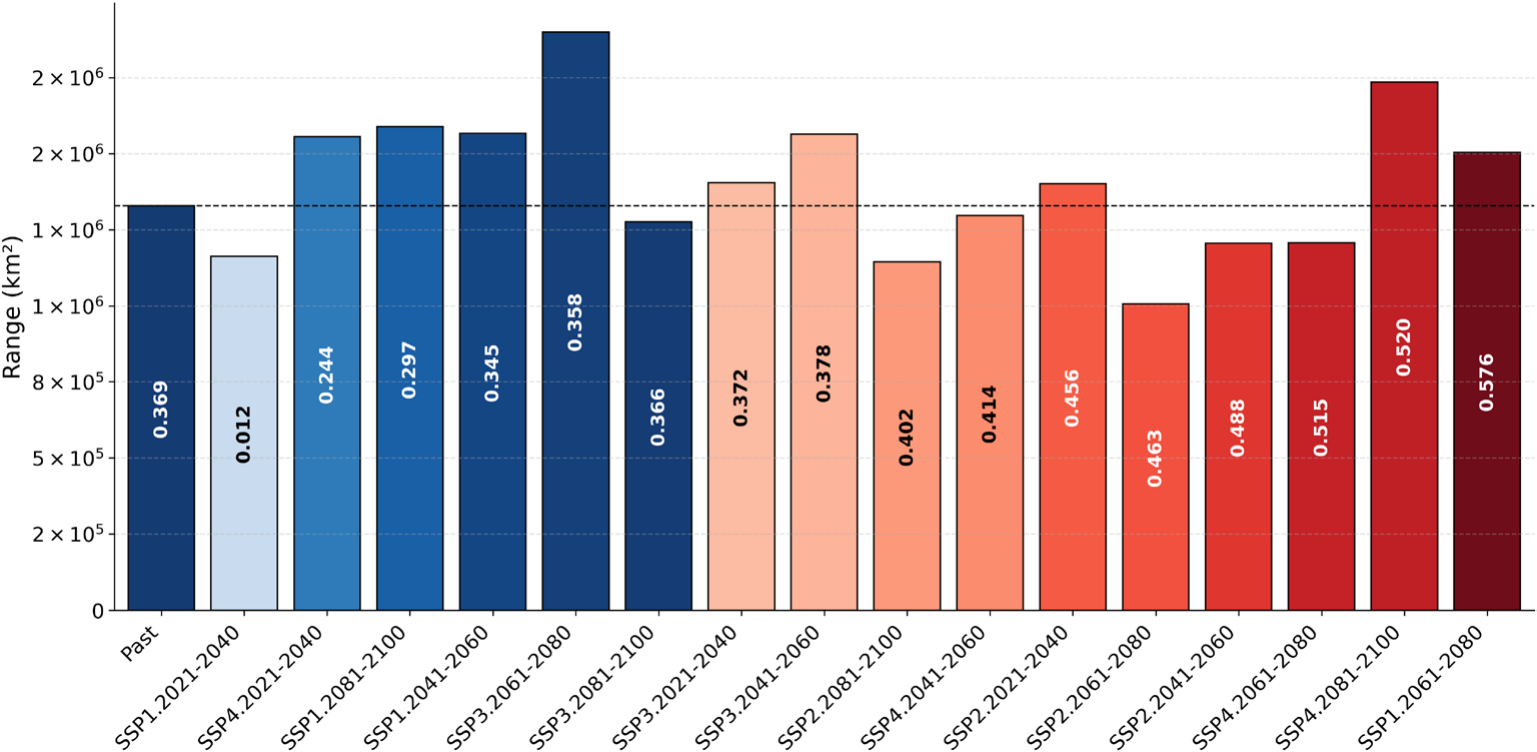
Range (km^2^) of *Aedes albopictus* for the past and future scenarios. Bars show the total range area; numeric labels inside bars are the corresponding Composite Index Scale (CIS) values. Bars are ordered with Past first, followed by all futures sorted by ascending CIS values. Colors encode CIS relative to the Past CIS (threshold): Blue shades for CIS ≤ Past CIS and red shades for CIS > Past CIS, with darker tones indicating values farther from the threshold (higher CIS = higher risk). A thin dashed horizontal line indicates the past range to facilitate comparison.

In contrast, for *A. albopictus* , a comparison of CIS values between past and future scenarios revealed that 10 scenarios exhibited improved habitat quality, with CIS values greater than the past CIS value of 0.369. These scenarios include SSP1 (2061–2080), SSP2 across all time periods (2021–2040, 2041–2060, 2061–2080, 2081–2100), SSP3 across two time periods (2021–2040, 2041–2060), and SSP4 across three time periods (2041–2060, 2061–2080, 2081–2100) (Figure 6). Among these improved habitat quality scenarios, five scenarios demonstrated an increase in future ranges along with improved habitat quality, which indicates greater vulnerability to dengue transmission. These scenarios are SSP1 (2061–2080), SSP2 (2021–2040), SSP3 (2021–2040), SSP3 (2041–2060), and SSP4 (2081–2100). However, six scenarios did not exhibit good habitat quality. These scenarios include SSP1 (2021–2040, 2041–2060, 2081–2100), SSP3 (2061–2080, 2081–2100), and SSP4 (2021–2040) (Figure 6). Among these six scenarios, four (SSP1: 2041–2060, 2081–2100; SSP3: 2061–2080; SSP4: 2021−2040) showed an increase in future ranges, but their CIS values remained below 0.369, indicating poorer habitat quality compared to the past scenario (Figure 6).

### 3.4 Correlation of Vulnerable Pixels and CIS Values

**FIGURE 7:**
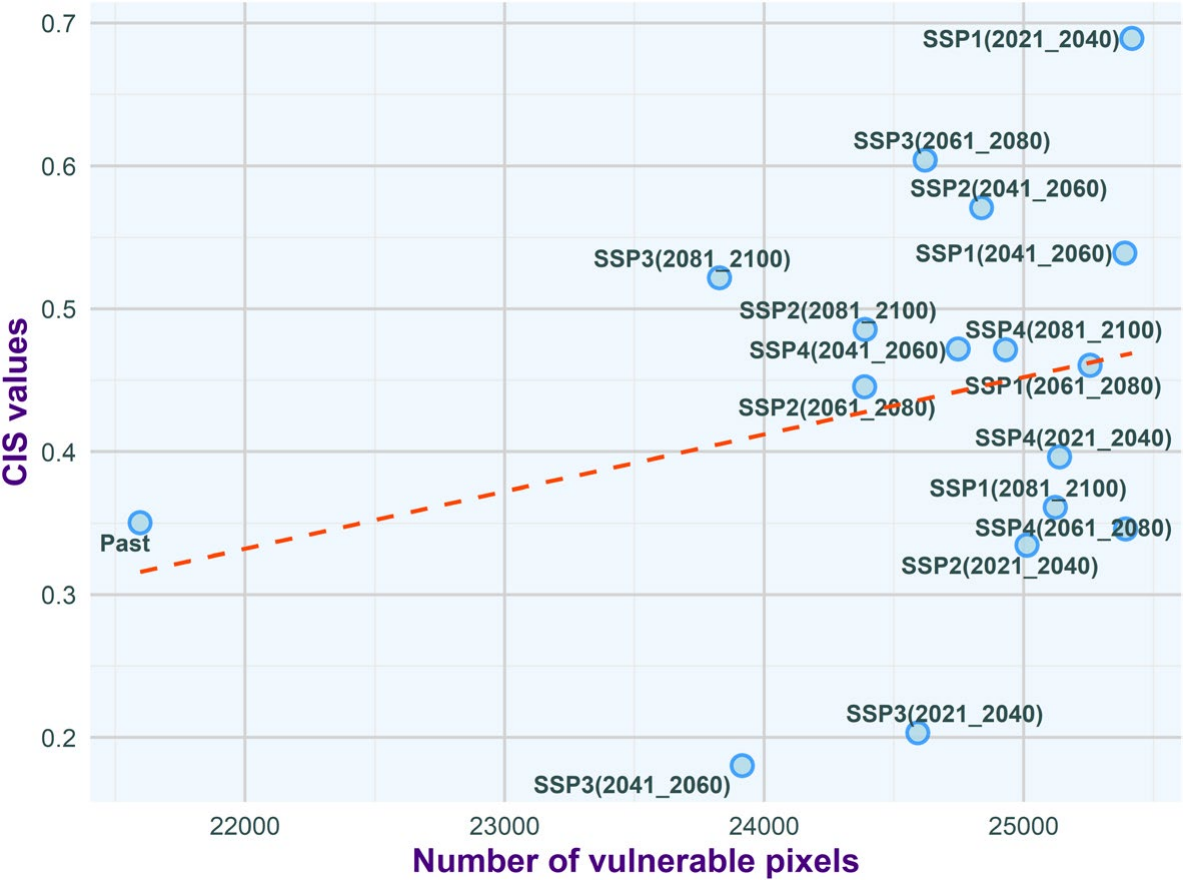
Correlation between the number of vulnerable pixels within the range of a scenario and the Composite Index Scale (CIS) value for *Aedes aegypti* . Here, vulnerable pixels are defined as those with habitat probability values equal to or greater than 0.5. The orange line indicates the regression line.

**FIGURE 8:**
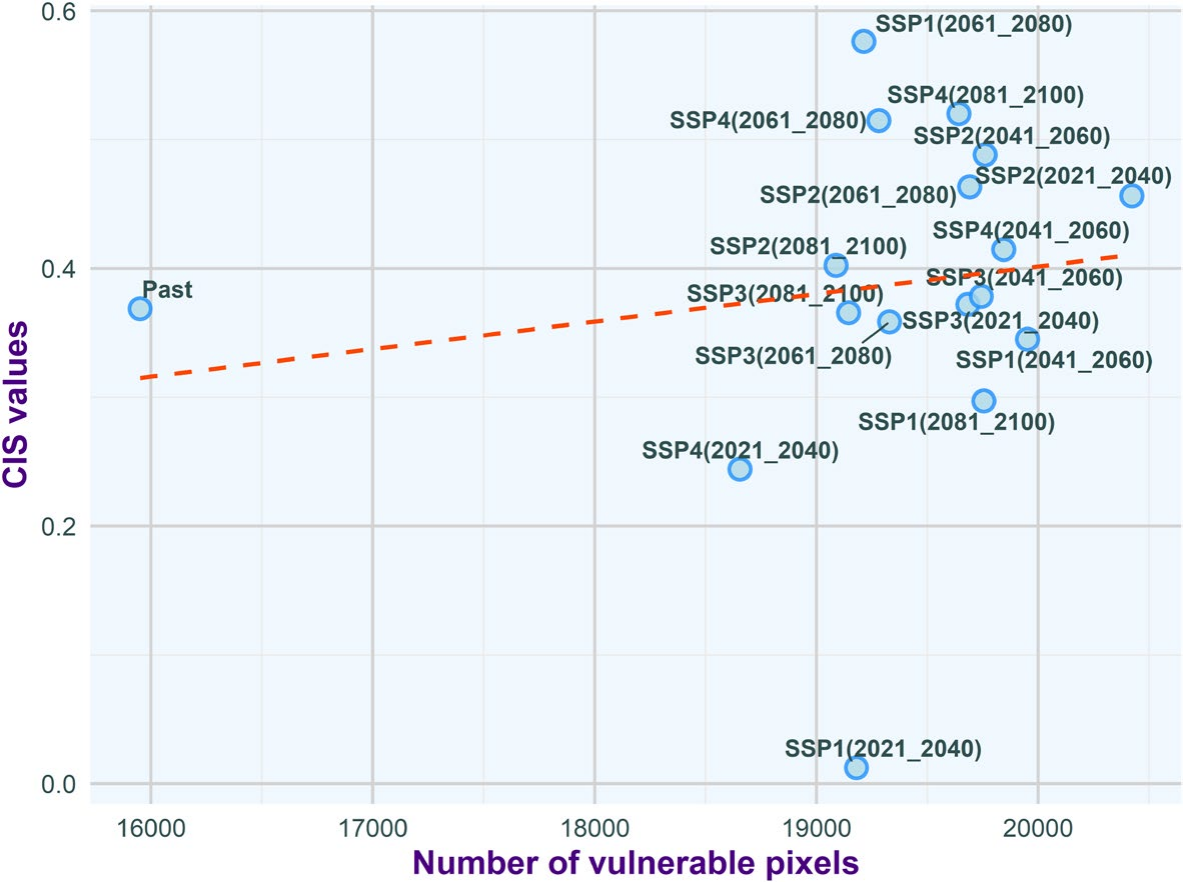
Correlation between the number of vulnerable pixels within the range of a scenario and the Composite Index Scale (CIS) value for *Aedes albopictus* . Here, vulnerable pixels are defined as those with habitat probability values equal to or greater than 0.5. The orange line indicates the regression line.

A correlation was observed between the number of vulnerable pixels (defined as those with habitat probability ≥ 0.5) and CIS values for both species. This relationship highlights the effectiveness of CIS as an indicator of habitat quality, suggesting that an increase in vulnerable pixels is associated with higher CIS values. This correlation emphasizes the heightened risk of dengue transmission in these regions (Figures 7 and 8).

Cross‐validation confirmed the stability of CIS across data partitions. The MaxEnt models yielded consistently high performance (mean AUC = 0.92 ± 0.001; mean TSS = 0.788 ± 0.026). Correspondingly, CIS values derived from each fold showed minimal variation, indicating that the scale is robust and not an artifact of overfitting. These results suggest that CIS reliably integrates multiple habitat quality components beyond raw suitability.

## 4 Discussion

The AUC values (> 0.9) of the MaxEnt model for both species indicate a high level of accuracy in predicting their spatial distribution across different scenarios. The Jackknife analysis identified various bioclimatic variables with differing contribution levels that drive the distributions of *A. aegypti* and *A. albopictus* (Figures 2, 3, 4). Both species exhibited distinct preferences for their bioclimatic habitats; however, these findings are consistent with previous research. For instance, *A. aegypti* demonstrated the highest contribution in most models for bio11, indicating its preference for warmer climates (Kraemer et al. 2019). Additionally, significant contributions were also observed in other scenarios involving bio13, which suggests that this species requires abundant water sources for breeding habitats (Brady et al. 2014). In comparison, *A. albopictus* displayed a stronger response to precipitation‐related variables, particularly bio16, indicating its greater tolerance for temperate and tropical regions (Medley et al. 2019). These results underscore the importance of bioclimatic factors as key determinants of distribution for both species, highlighting their ecological adaptability and potential for range expansion in response to changing climatic conditions.

The shift in habitat vulnerability predicted by our modeling indicates an increase in suitable areas for both species in the future. For instance, the habitat vulnerability for *A. aegypti* is projected to increase to 2,220,554 km^2^, whereas for *A. albopictus* , this increase is expected to reach 1,734,846 km^2^. These increases in vulnerability are supported by previous studies (Aliaga‐Samanez et al. 2024; Jing et al. 2024; Meena et al. 2024; Prasad 2024). The results indicate that Thailand is currently the most vulnerable country for *A. aegypti* ; however, projections suggest that India will surpass Thailand in vulnerability in the future. For *A. albopictus* , historically, China has been identified as the most vulnerable country for this species, but projections showed that Indonesia will become the most vulnerable. This shift may be attributed to environmental and demographic changes, which could ultimately enhance vector prevalence in the region.

As previously discussed, an increase in habitat range does not necessarily imply that the expanded range is suitable for the establishment of mosquito vectors or an increase in the threat of dengue disease. Therefore, we should accept that habitat quality is crucial for understanding the implications of range expansion. We assessed future habitat quality using our CIS. This assessment indicates that *A. aegypti* is likely to encounter increasingly favorable conditions that could support dengue transmission under future scenarios. Among the 16 future scenarios analyzed, 12 scenarios indicated improved habitat quality, and 10 of these showed an increase in future range, which is more vulnerable. Two scenarios demonstrated improved habitat quality despite a decrease in future ranges: SSP3 (2061–2080) with a CIS value of 0.604, which is greater than the past value of 0.350, and SSP4 (2041–2060) with a CIS value of 0.472. Conversely, four scenarios—SSP2 (2021–2040), SSP3 (2021–2040), SSP3 (2041–2060), and SSP4 (2061–2080)—showed an increase in ranges compared to past scenarios; however, these ranges were of poor quality, as indicated by CIS values less than 0.350 (Figure 5). These results highlight a fundamental limitation of traditional SDMs and risk maps, which primarily emphasize climatic suitability and range size without accounting for habitat quality. By integrating habitat persistence, niche breadth, and stability into a composite measure, CIS advances beyond standard MaxEnt outputs by distinguishing between transient expansions and genuinely high‐quality habitats. This distinction is critical for more accurate dengue risk predictions, as it identifies areas of persistent vulnerability rather than temporary climatic opportunities.

A potential concern with the CIS is the risk of circularity if validation relies only on correlations with raw model outputs. To address this, we re‐ran the species distribution models using 5‐fold cross‐validation, which allowed CIS to be evaluated against withheld test data rather than the training subsets. The consistently high predictive performance across folds and the stability of CIS values support its robustness as a measure of habitat quality. Although epidemiological dengue outbreak data would provide the most direct form of independent validation, such datasets were not available for the spatial and temporal scales of this study. We therefore consider cross‐validation to represent a rigorous internal test of CIS reliability. Future studies that integrate CIS with independent ecological or epidemiological outcomes will be valuable for further strengthening its validity.

Similarly, for *A. albopictus* , our analysis of habitat quality assessment indicates that this species has also shifted towards improved habitat quality in conjunction with range increases in some scenarios (Figure 6). This shift in range is consistent with previous findings, which suggest that *A. albopictus* prefers new ranges with favorable climate conditions (Abbasi 2025b; Brady et al. 2014; Medley et al. 2019; Prasad 2024). However, in some cases, the range of this species is increasing, but the habitat quality is not good. These contrasting results show the complexity of habitat dynamics, and they suggest that although the range has expanded, it did not necessarily equate to improved suitability for *A. albopictus*.

In comparing the risks posed by both species of *Aedes*, our results reveal a dual nature of habitat suitability and disease transmission risk for both species. For *A. aegypti* , the majority of future scenarios indicate a strong association between improved habitat quality and a heightened risk of disease transmission. In contrast, *A. albopictus* presents a more complex scenario: although some scenarios show improved habitat quality, others indicate suboptimal conditions. This complexity underscores the necessity for targeted vector control measures tailored to each species.

Public health strategies must consider these dynamics to effectively mitigate the risks associated with increased dengue transmission, particularly in regions projected to experience improvements in habitat quality for both *A. aegypti* and *A. albopictus* . Understanding these interactions is vital for developing adaptive management approaches in response to climate change and urbanization.

## 5 Conclusion

Our study highlights the importance of habitat quality risk assessment for mosquito vector‐borne diseases, emphasizing its necessity over traditional range‐based risk assessments. Integrating the newly developed CIS scale with species distribution modeling projections informs not only the spatial distribution dynamics of species but also the ecological capacity of new ranges to support stable mosquito populations. By integrating habitat quality metrics with climatic suitability, the CIS advances beyond traditional SDMs, offering a more realistic and actionable tool for anticipating dengue transmission risk in a changing climate. The consistent alignment of CIS values with habitat suitability and vulnerability underscores the strength of the CIS index. Future efforts should focus on incorporating CIS parameters to study habitat quality at local or fine scales.

## Author Contributions

**Muhammad Naeem:** conceptualization (equal), data curation (equal), formal analysis (equal), methodology (equal), software (equal), validation (equal), writing – original draft (equal). **Lei Zhu:** conceptualization (equal), methodology (equal), writing – original draft (equal). **Nawaz Haider Bashir:** conceptualization (equal), validation (equal), writing – review and editing (equal). **Maryam Riasat:** data curation (equal), investigation (equal), writing – review and editing (equal). **Wenbo Li:** data curation (equal), project administration (equal), writing – review and editing (equal). **Huanhuan Chen:** conceptualization (equal), funding acquisition (equal), project administration (equal), supervision (equal), writing – review and editing (equal).

## Conflicts of Interest

The authors declare no conflicts of interest.

## Supporting information


**Table S1:** Collections.

**Table S2:** CIS parameters *Aedes aegypti* .

**Table S3:** CIS parameters *Aedes albopictus* .

## Data Availability

The spatial distribution data of *Aedes* species were from the Global Biodiversity Information Facility (GBIF; https://www.gbif.org/), and some specimens were collected from Pakistan by us, which are included in Supporting Informations. Climate data were obtained from Worldclim (http://www.worldclim.org).
